# Excitability and Synaptic Alterations in the Cerebellum of APP/PS1 Mice

**DOI:** 10.1371/journal.pone.0034726

**Published:** 2012-04-12

**Authors:** Eriola Hoxha, Enrica Boda, Francesca Montarolo, Roberta Parolisi, Filippo Tempia

**Affiliations:** 1 Neuroscience Institute Cavalieri Ottolenghi (NICO), University of Turin, Turin, Italy; 2 National Institute of Neuroscience-Italy (INN), University of Turin, Turin Italy; University of North Dakota, United States of America

## Abstract

In Alzheimer's disease (AD), the severity of cognitive symptoms is better correlated with the levels of soluble amyloid-beta (Aβ) rather than with the deposition of fibrillar Aβ in amyloid plaques. In APP/PS1 mice, a murine model of AD, at 8 months of age the cerebellum is devoid of fibrillar Aβ, but dosage of soluble Aβ_1–42_, the form which is more prone to aggregation, showed higher levels in this structure than in the forebrain. Aim of this study was to investigate the alterations of intrinsic membrane properties and of synaptic inputs in Purkinje cells (PCs) of the cerebellum, where only soluble Aβ is present. PCs were recorded by whole-cell patch-clamp in cerebellar slices from wild-type and APP/PS1 mice. In APP/PS1 PCs, evoked action potential discharge showed enhanced frequency adaptation and larger afterhyperpolarizations, indicating a reduction of the intrinsic membrane excitability. In the miniature GABAergic postsynaptic currents, the largest events were absent in APP/PS1 mice and the interspike intervals distribution was shifted to the left, but the mean amplitude and frequency were normal. The ryanodine-sensitive multivescicular release was not altered and the postsynaptic responsiveness to a GABA_A_ agonist was intact. Climbing fiber postsynaptic currents were normal but their short-term plasticity was reduced in a time window of 100–800 ms. Parallel fiber postsynaptic currents and their short-term plasticity were normal. These results indicate that, in the cerebellar cortex, chronically elevated levels of soluble Aβ_1–42_ are associated with alterations of the intrinsic excitability of PCs and with alterations of the release of GABA from interneurons and of glutamate from climbing fibers, while the release of glutamate from parallel fibers and all postsynaptic mechanisms are preserved. Thus, soluble Aβ_1–42_ causes, in PCs, multiple functional alterations, including an impairment of intrinsic membrane properties and synapse-specific deficits, with differential consequences even in different subtypes of glutamatergic synapses.

## Introduction

AD is a neurodegenerative disorder characterized by a progressive decline in cognitive brain functions. The pathological hallmarks of AD are parenchymal plaques containing Aβ proteins and intraneuronal neurofibrillary tangles. The hypothesis that Aβ aggregates play a causal role in AD [1] is supported by several lines of evidence (reviewed in [2]). However, while in AD patients the fibrillar plaque density is weakly correlated with the severity of dementia and the extent of synaptic loss [3]–[4], these parameters show a strong correlation with the levels of soluble aggregates of Aβ [5]–[7]. Soluble Aβ has toxic effects on synaptic function and it can readily diffuse in the extracellular spaces, as shown by the fact that intracerebroventricular injections are effective in blocking synaptic plasticity [8]. In this research we utilize a transgenic murine model of AD, the APP/PS1 mouse [9], which bears a marked forebrain amyloidosis while hindbrain structures, including the cerebellum, are devoid of fibrillar Aβ plaques. In APP/PS1 mice, the first amyloid plaques appear in the cerebral cortex at 6 weeks of age [9]. In hippocampus, amyloid deposition starts in the dentate gyrus at 2–3 months of age and in CA1 at 4–5 months [9]. In striatum, thalamus and brain stem, amyloidosis appears between 3 and 5 months of age [9]. At the age of 8 months the entire forebrain is covered with amyloid [9], while in the cerebellum no amyloid plaques are present (unpublished observations). In spite of the lack of amyloid deposits, the cerebellum of APP/PS1 mice might be reached by significant levels of soluble Aβ via the extracellular spaces, as confirmed by the high amount of soluble Aβ_1–42_ observed in the present study. Actually, neurodegeneration in AD patients does not affect only the cerebrum but also the cerebellum, although generally in later stages of the disease. Many lines of research have shown that, in AD patients, the cerebellum is reduced in volume in a similar fashion as cerebral hemispheres [10]–[11], the Purkinje cell (PC) number is decreased [12] and the levels of Aβ_1–42_ are increased more than twofold relative to controls [13].

The best characterized effects of soluble Aβ on neuronal function are exerted on glutamatergic synaptic transmission, although with variable results in different experimental models [8], [14]–[18] (reviewed in [2]). In contrast to a decrease of network activity predicted from the glutamatergic hypothesis, neuronal hyperactivity has been reported in the hippocampal-entorhinal cortex network [19]–[21] and in cerebral cortex [22]. Such hyperactivity has been attributed either to intrinsic hyperexcitability [20]–[21] or to reduced inhibition [22]. Therefore, it is important to simultaneously consider the effects of Aβ on intrinsic excitability, glutamatergic and GABAergic synaptic transmission.

In the present study, we investigated the alterations in intrinsic excitability and synaptic transmission in the cerebellar PCs. We show, in the cerebellar cortex of APP/PS1 mice, a reduction of intrinsic membrane excitability of PCs and alterations of the release of GABA from interneurons and of glutamate from CFs, while the release of glutamate from PFs and all postsynaptic mechanisms are preserved. These results indicate that, in the cerebellar cortex, elevated levels of soluble Aβ are associated with alterations of the intrinsic excitability of PCs and of the function of specific glutamatergic and GABAergic presynaptic terminals.

## Results

### Beta-amyloid levels in cerebellar extracellular fluids

The *APP* and *PS1* transgenes of APP/PS1 mice are transcribed under the control of the neuron-specific *Thy1* promoter, which is abundantly expressed in the forebrain but much less in the cerebellum [9]. However, soluble forms of A are known to freely diffuse in the extracellular fluids and distribute in all communicating compartments, including adjacent regions like forebrain and hindbrain. We performed ELISA assays in order to compare the abundance of soluble Aβ in the cerebellum relative to the forebrain, where most of the transgene transcription occurs. At two months of age, when amyloid plaque formation is still negligible (Radde et al, 2006; and data not shown), the levels of soluble Aβ_1–42_ in the cerebellum were 51.2% when compared to the forebrain (taken as 100%). At 8 months, which corresponds to the age at which most of the results of the following experiments were obtained, the cerebellar amount of soluble Aβ_1–42_ increased to 140.5% relative to the forebrain.

### Intrinsic membrane properties and excitability of PCs

In cerebellar slices from mice of 7–8 months of age, the spontaneous discharge of PCs was recorded immediately following the achievement of the whole-cell configuration, before application of synaptic blockers. The majority of PCs spontaneously fired action potentials (12 out of 14 wild-type and 16 out 20 APP/PS1 cells). The mean firing frequency of PCs was 36.8±5.6 spikes/sec for wild-type and 26.4±5.0/sec for APP/PS1, without a significant difference (t-test, P>0.05).

**Figure 1:**
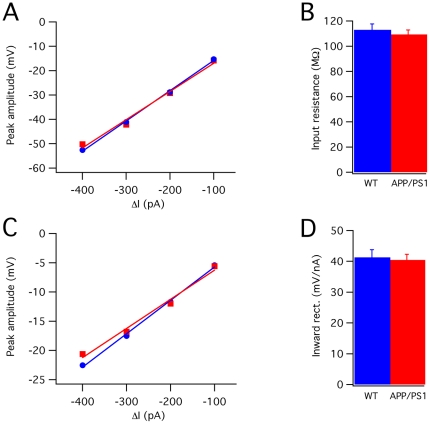
Responses to hyperpolarizing currents. (**A**) Example of plots of peak amplitudes versus injected currents. Each point is the average of five trials. Data were fitted by a linear function. Blue circles and lines: wild-type; red squares and lines: APP/PS1 (**B**) Mean input resistance values of wild-type (blue column, n = 14) and APP/PS1 (red column, n = 20) PCs. There is no significant difference (P>0.05). (**C**) Example of plots of inward rectification (IR) versus injected currents. Each point is the average of five trials. The line is the linear fitting. (**D**) Mean inward rectification of wild-type and APP/PS1 PCs.

Passive membrane properties of wild-type (n = 14 cells) and APP/PS1 PCs (n = 20 cells) were assessed by delivering hyperpolarizing current steps in the presence of blockers of ionotropic glutamate and GABA_A_ receptors. The input resistance was 112.9±4.7 MΩ in wild-type and 109.4±3.6 MΩ in APP/PS1 PCs (Fig. 1A–B; Student's t-test: P>0.05). In the responses to hyperpolarizing steps, the amplitude of the bump due to the *I*_H_ inward rectifier current was 41.3±2.5 mV in wild-type and 40.4±1.8 mV in APP/PS1, with no significant difference (Fig. 1C–D; Student's t-test: P>0.05).

**Figure 2:**
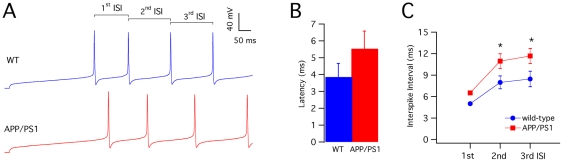
Evoked firing properties of APP/PS1 PCs. (**A**) Wild-type (blue) and APP/PS1 (red) PC evoked action potential firing. In the wild-type trace, the first three interspike intervals (ISI) are shown. (**B**) Mean latency of the first spike for PCs from wild-type (n = 14) and APP/PS1 (n = 20). The difference is not significant (P>0.05). (**C**) Plot of the first three ISIs of wild-type (n = 14) and APP/PS1 (n = 20) mice PCs. The second and third ISIs are significantly prolonged in APP/PS1 mice (*p<0.05, t test).

Membrane excitability and active membrane properties were assessed by delivering, starting from a *V*_m_ close to −70 mV, depolarizing current steps, which elicited repetitive firing in all PCs of both groups of mice (Fig. 2A). The latency of the first spike was highly variable and displayed a nonsignificant tendency to longer values in APP/PS1 relative to wild-type PCs (MW test: P>0.05, Fig. 2B). The first interspike interval was slightly prolonged in APP/PS1 PCs, although the difference was not significant (wild-type: 5.0±0.2; APP/PS1 6.5±0.1; Student's t-test: P>0.05, Fig. 2C). However, APP/PS1 PCs displayed significantly prolonged second (wild-type: 8.0±0.9 ms; APP/PS1: 11.0±1.0 ms; Student's t-test: P<0.05) and third (wild-type: 8.5±1.1 ms; APP/PS1: 11.7±1.1 ms; Student's t-test: P<0.05) interspike intervals, indicating a more pronounced frequency adaptation (Fig. 2C).

**Figure 3:**
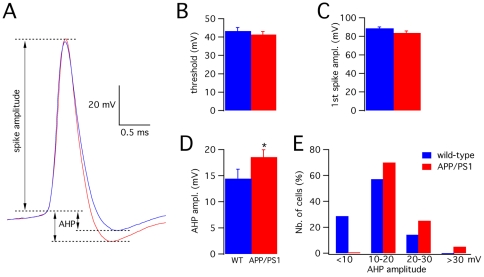
Evoked action potential properties. (**A**) Superimposed traces of first action potentials of wild-type (blue) and APP/PS1 (red) PCs. The horizontal dotted lines represent the threshold and the negative peaks reached by the afterhyperpolarization (AHP). The vertical arrows indicate the measurements of spike amplitude (for both wild type and APP/PS1) and of the AHP (separately for wild-type and APP/PS1). (**B**) Mean action potential threshold (P>0.05). (**C**) Mean amplitude of the first spike (P>0.05). (**D**) Mean AHP amplitude in wild-type (n = 14) and APP/PS1 PCs (n = 20). The difference is statistically significant (*: p<0.05, t test). (**E**) Histogram of AHP sizes divided in four groups (<10, 10–20, 20–30, >30 mV). Note the shift to the right of the distribution in APP/PS1 mice.

The threshold and the amplitude of the first action potential were comparable in PCs for both groups (Student's t-test: P>0.05, Fig. 3A–C). In contrast, the fast afterhyperpolarization (AHP) following the first action potential was significantly larger in APP/PS1 (18.6±1.4 mV) relative to wild-type PCs (14.4±1.8 mV; MW test: P<0.025, Fig. 3D; representative traces are shown in panel A). The distribution of AHP amplitudes was clearly shifted to higher values in APP/PS1 mice (Fig. 3E), confirming an overall tendency to larger AHPs.

In order to assess whether these alterations of membrane excitability were due to the production of Aβ rather than to some other unspecific process, we have repeated the analyses on young APP/PS1 (n = 10) and wild-type (n = 8) mice at the age of two months. In fact, in two-months-old APP/PS1 mice there is no significant amyloid formation or deposition (Radde et al., 2006; and data not shown). The input resistance of PCs was 88.1±4.9 MΩ in wild-type and 93.4±4.9 MΩ in APP/PS1 mice (P>0.05). In the responses to hyperpolarizing steps, the amplitude of the bump due to the *I*_H_ inward rectifier current was 47.6±3.0 mV in wild-type and 54.5±4.3 mV in APP/PS1, with no significant difference (P>0.05).

In evoked action potential firing, the latency of the first spike was highly similar, with 12.2±3.7 ms in wild type versus 12.0±2.7 ms in APP/PS1 (P>0.05). The first interspike interval was 7.4±1.0 ms in wild-type and 8.2±0.7 in APP/PS1 (P>0.05). More importantly, there was no significant difference in the second interspike interval, with 9.5±1.2 ms in wild type and 11.7±2.0 in APP/PS1 PCs (P>0.05). The third interspike interval could not be analyzed because some PCs fired only three action potentials. The threshold and the amplitude of the first action potential were very similar in PCs of both groups (P>0.05). The fast afterhyperpolarization (AHP), which was significantly larger in older APP/PS1 mice, in PCs of two-months-old mice had the same amplitude as in age-matched wild type controls (wild type: 21.5±2.3; APP/PS1: 18.4±1.8; P>0.05). Therefore, no alterations of membrane excitability were present in young adult mice (2 months old), in which the production and deposition of Aβ is not yet significant. In contrast, PCs from older adult (7–8 months old) APP/PS1 mice displayed a reduction of excitability accompanied by an increased size of the AHP. Table 1 contains a comprehensive list of all membrane parameters analyzed in wild-type and APP/PS1 PCs

**Table 1:** Comparison of passive and active membrane properties of wild-type versus APP/PS1 mice of 8 and of 2 months of age.

	Spontaneous firing frequency without blockers (Hz)	Input resistance (MΩ)	*I*_H_ –dependent voltage deflection (mV)	First spike latency (ms)	1^st^ ISI (ms)	2^nd^ ISI (ms)	3^rd^ ISI (ms)	Threshold (mV)	1^st^ AP amplitude (mV)	AHP amplitude (mV)
Wild-type 8 months n = 14	36.8±5.6	112.9±4.7	41.3±2.5	3.9±0.8	5.0±0.2	8.0±0.9	8.5±1.1	43.3±1.9	88.6±1.6	14.4±1.8
APP/PS1 8 months n = 20	26.4±5.0	109.4±3.6	40.4±1.8	5.5±1.1	6.5±0.1	11.0±1.0	11.7±1.1	41.3±1.6	83.6±2.2	18.6±1.4
P	n.s.	n.s.	n.s.	n.s.	n.s.	<0.05	<0.05	n.s.	n.s.	<0.025
Wild-type 2 months n = 8	43.9±20.0	88.1±4.9	47.6±3.0	12.2±3.7	7.4±1.0	9.5±1.2	n.a.	52.0±3.1	82.7±4.1	21.5±2.3
APP/PS1 2 months n = 10	45.1±13.6	93.4±4.9	54.5±4.3	12.0±2.7	8.2±0.7	11.7±2.0	n.a.	54.7±2.0	82.3±2.2	18.4±1.8
P	n.s.	n.s.	n.s.	n.s.	n.s.	n.s.	n.a.	n.s.	n.s.	n.s.

### Functional analysis of GABAergic synapses onto PCs

**Figure 4:**
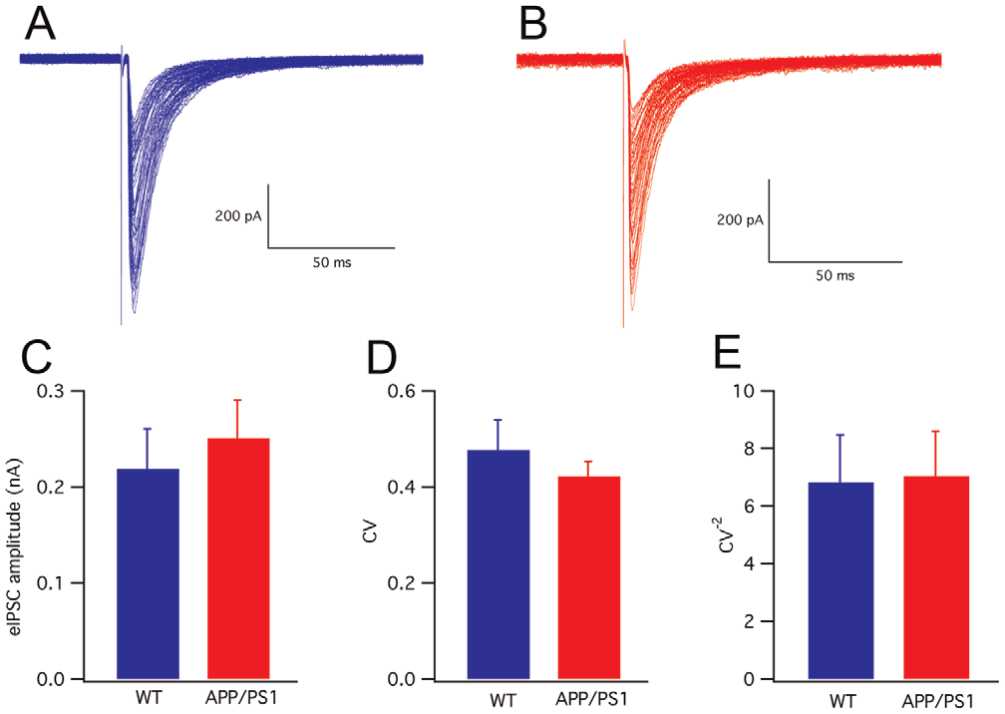
Evoked inhibitory post-synaptic currents (eIPSCs) in wild-type and APP/PS1 PCs. (A) Representative recordings of eIPSCs in wild-type and (B) APP/PS1 mice. The number of superimposed traces is 65 in A and 70 in B. There is no significant difference (number of cells n = 10 for wild-type, n = 11 for APP/PS1; P>0.05) between the two groups in either the amplitude (C) or the coefficient of variation (D) or the CV^−2^ (E).

Evoked inhibitory postsynaptic currents (eIPSCs), recorded in PCs during block of ionotropic glutamate receptors, displayed no significant difference between wild-type and APP/PS1 mice (wild-type: 218.6±41.8 pA, n = 10; APP/PS1: 250.8±39.4 pA, n = 11; P>0.05). Also the coefficient of variation (CV) and the value of CV^−2^ were not significantly different (P>0.05; Fig. 4). The eIPSCs of both wild-type and APP/PS1 PCs were completely blocked by application of a GABA_A_ receptor antagonist (gabazine, 20 µM, data not shown).

**Figure 5:**
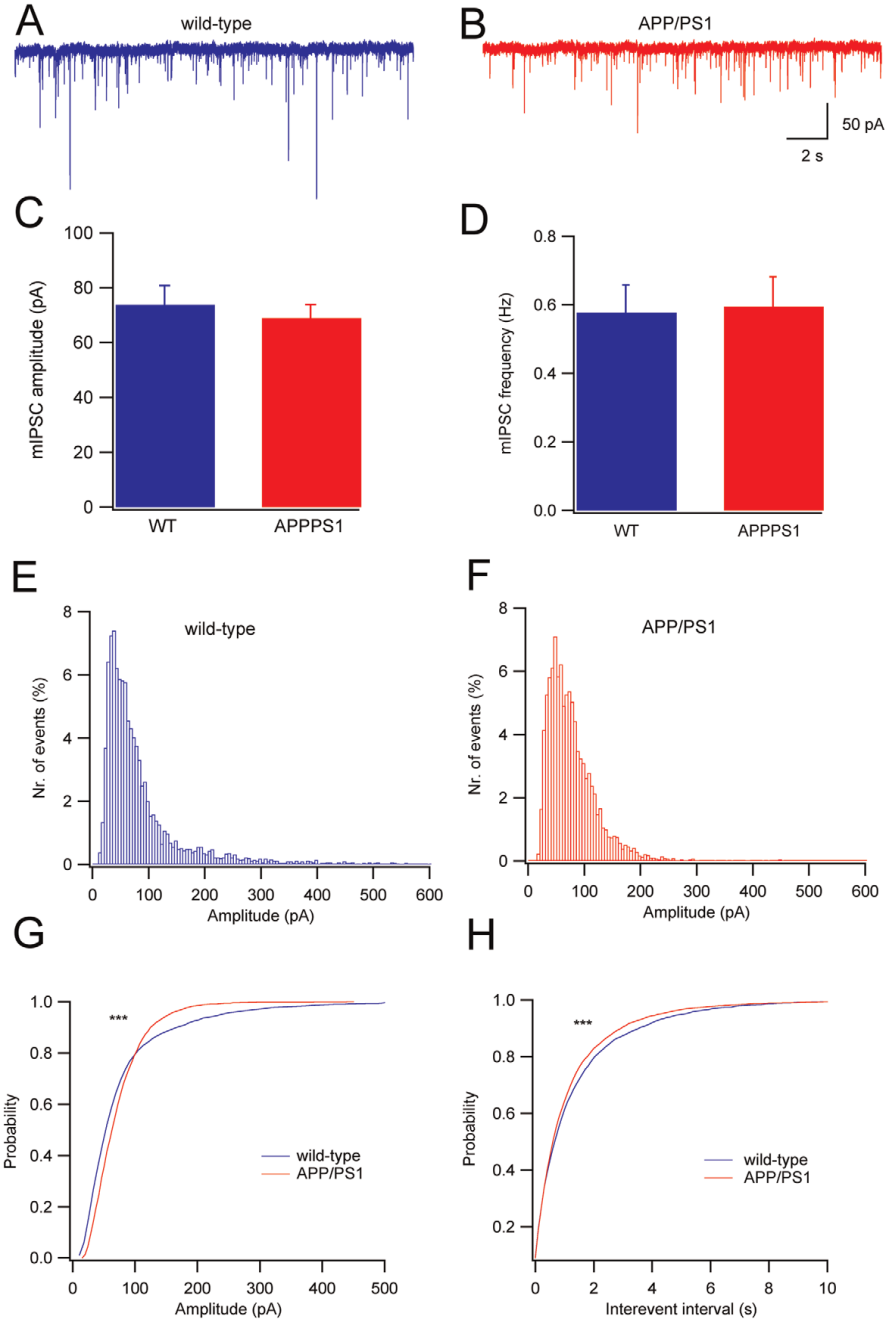
Miniature inhibitory post-synaptic currents (mIPSCs) in wild-type and APP/PS1 PCs. (**A**) Representative recordings of spontaneous mIPSCs of a wild-type and (**B**) an APP/PS1 PC. Mean amplitude (**C**) and frequency (**D**) of mIPSCs (P>0.05 for both). (**E**) Amplitude distribution of miniature GABAergic events in wild-type (n = 15 cells) and (**F**) APP/PS1 (n = 18 cells) mice. (**G**) Comparison of the cumulative distributions of amplitudes of the two groups. Note the selective loss of large amplitude mIPSCs in APP/PS1 PCs (***P<0.001, KS test). (**H**) Cumulative frequency plot for inter-event intervals between the two groups (***P<0.001, KS test).

Miniature inhibitory, GABAergic, currents (mIPSCs) were isolated by the application of ionotropic glutamate receptor blockers and TTX (Fig. 5 A–B). Under these conditions, the residual miniature currents were completely abolished by gabazine (data not shown). The mean amplitude of mIPSCs showed no significant difference between wild-type and APP/PS1 mice (wild-type: 73.8±7.0 pA, n = 24; APP/PS1: 68.9±4.9 pA, n = 31; Student's t-test, P>0.05; Fig. 5C). The amplitude histograms had a peak at 30–50 pA and were skewed, with a tail on the right, representing the largest events (Fig. 5E–F). Such a tail was clearly shorter in APP/PS1 PCs relative to wild-type, indicating that large mIPSCs were underrepresented, as also shown by the cumulative plot (Fig. 5G). The comparison of the cumulative distributions (Fig. 5G) revealed a significant difference (KS test: P<0.001), which can be attributed to the underrepresentation of the largest events in APP/PS1 mice.

The mean frequency of mIPSCs was not different in the two groups of mice (wild-type: 0.578±0.080 Hz, n = 24; APP/PS1: 0.595±0.086 Hz, n = 31; Fig. 5D). However, the comparison of cumulative distributions for mIPSC interevent intervals revealed a significant difference between the two groups (Fig. 5H, KS test, P<0.001), with shorter intervals in APP/PS1 PCs. Rise time and decay kinetics of mIPSCs were not significantly different between wild-type and APP/PS1 PCs (KS test: P>0.05 data not shown).

**Figure 6:**
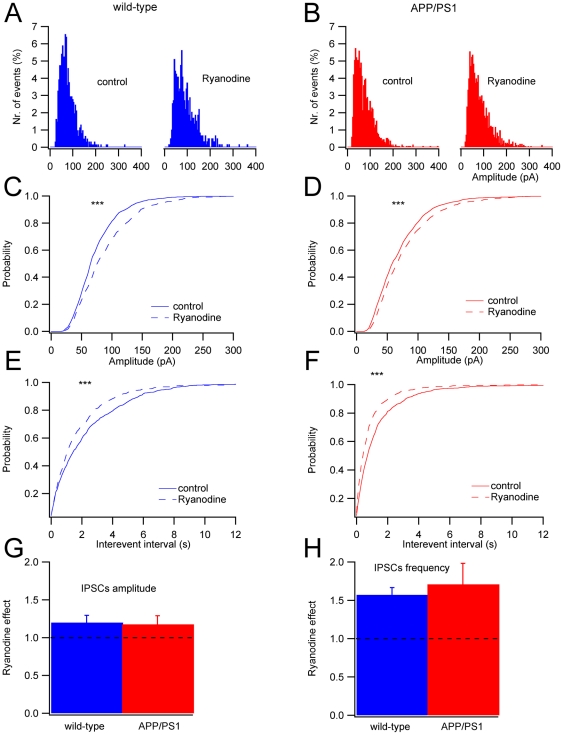
Ryanodine effects on mIPSCs. (**A**) mIPSCs amplitude histograms obtained in control condition and in the presence of ryanodine for wild-type (n = 6) and (**B**) for APP/PS1 (n = 13) cells. (**C**) Normalized cumulative amplitude histograms for wild-type and (**D**) for APP/PS1 cells (***P<0.001, KS test). (**E**) Normalized cumulative frequency plot for inter-event intervals for wild-type and (**F**) APP/PS1 cells (***P<0.001, KS test). (**G**) Ryanodine effect on the mIPSCs amplitude and (**H**) frequency for wild-type and APP/PS1 PCs. The effects of ryanodine relative to the controls before application are significant (P<0.01, Student's paired t-test) but no significant difference is present between genotypes.

Presynaptic ryanodine receptors (RyRs) have been shown to be involved in mIPSCs of PCs, where they are responsible for multivescicular release, generating large amplitude events [23]. To test whether the amplitude or frequency changes detected in APP/PS1 PCs were attributable to an alteration of RyRs, we recorded mIPSCs in the presence of ryanodine at a concentration (10 µM), which evokes Ca^2+^ release from intracellular stores [23]. The effects of ryanodine on mIPSCs were examined in n = 8 and n = 15 cells for wild-type and APP/PS1 mice respectively. Sample histograms are shown in Fig. 6A–B. In wild-type mice, six cells showed a significant (KS test: P<0.001; Fig. 6C, E) increase in amplitude and frequency. Also for APP/PS1 mice, 13 cells showed a significant (KS test: P<0.001; Fig. 6D, F) increase in frequency and amplitude. The effect of ryanodine was comparable for the two groups of animals both for amplitude (Fig. 6G; for wild-type normalized mean amplitude relative to control 1.20±0.10, n = 6; for APP/PS1 1.18±0.11, n = 13; Student's paired t-test, P<0.01) and frequency (Fig. 6H; for wild-type normalized mean frequency relative to control 1.57±0.09, n = 6; for APP/PS1 1.71±0.30, n = 13; Student's paired t-test, P<0.01). These results confirm that RyRs are functionally normal in APP/PS1 PCs.

**Figure 7:**
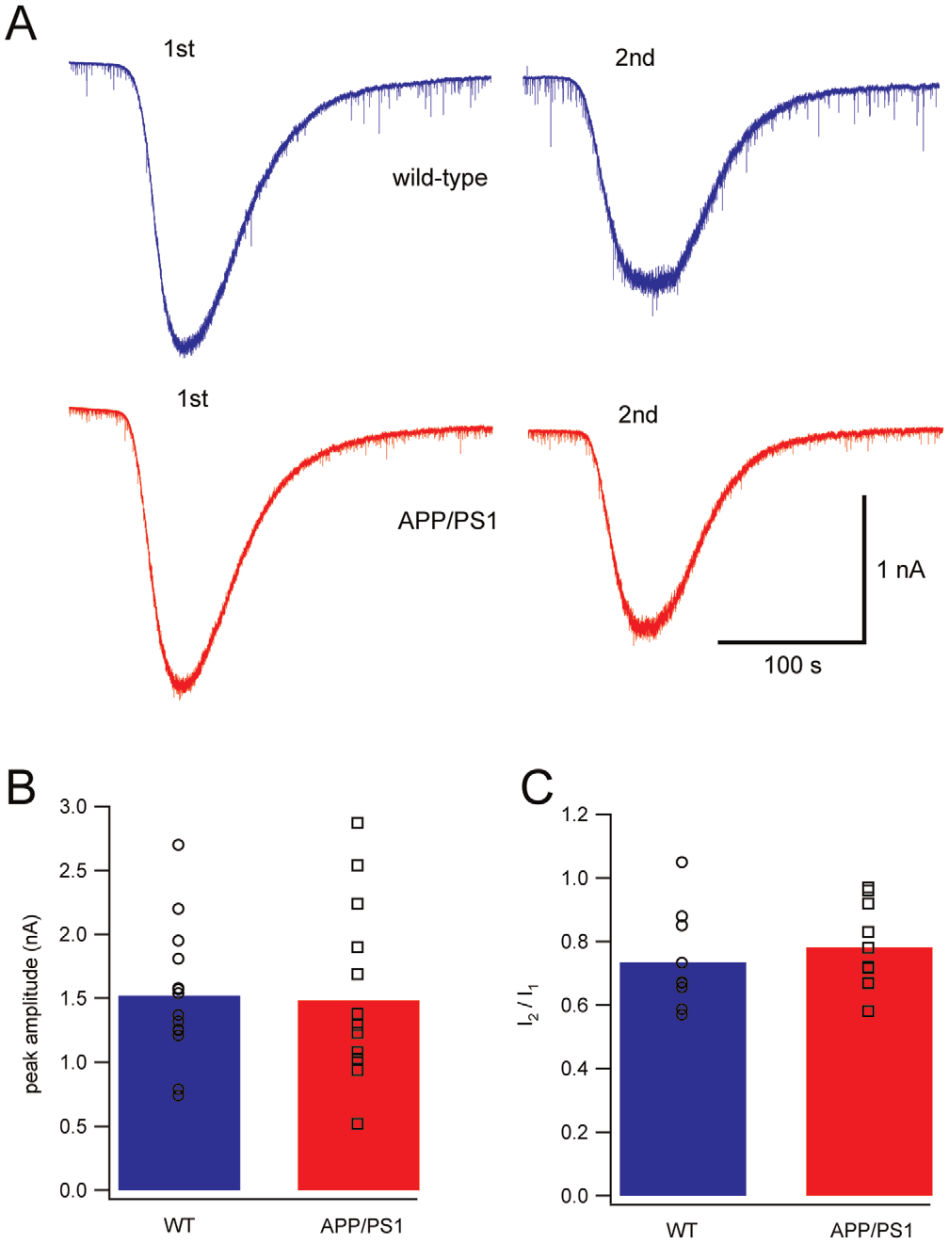
Responses to applications of muscimol. (**A**) Representative currents evoked by two applications of muscimol (0.5 µM), ten minutes apart, in wild-type (blue) and APP/PS1 cells (red). (**B**) Mean amplitude of peak currents in wild-type and APP/PS1 cells evoked by the first application of muscimol (P>0.05, Student's t-test). (**C**) Ratio of the second current peak amplitude relative to the first one for both groups (P>0.05, Student's t-test).

In order to assess whether, at the interneuron-PC synapse, postsynaptic alterations were also present, we monitored postsynaptic currents evoked by bath applications of the GABA_A_ receptor agonist muscimol (0.5 µM). Comparable postsynaptic currents were evoked by muscimol in wild-type and APP/PS1 PCs (Fig. 7A–B; mean amplitude for wild-type 1.52±0.14 nA, n = 14; for APP/PS1 1.48±0.18 nA, n = 14; Student's t-test, P>0.05). To test for a possible alteration of the desensitization properties of the GABA_A_ receptors, we applied muscimol again 10 minutes after the first application. The ratio between the two applications was comparable (Fig. 7C; wild-type 0.73±0.05, n = 10; APP/PS1 0.78±0.04, n = 11; Student's t-test, P>0.05), indicating that in APP/PS1 mice, PC GABA_A_ receptors have normal desensitization properties.

### Evoked excitatory postsynaptic currents in PCs

**Figure 8:**
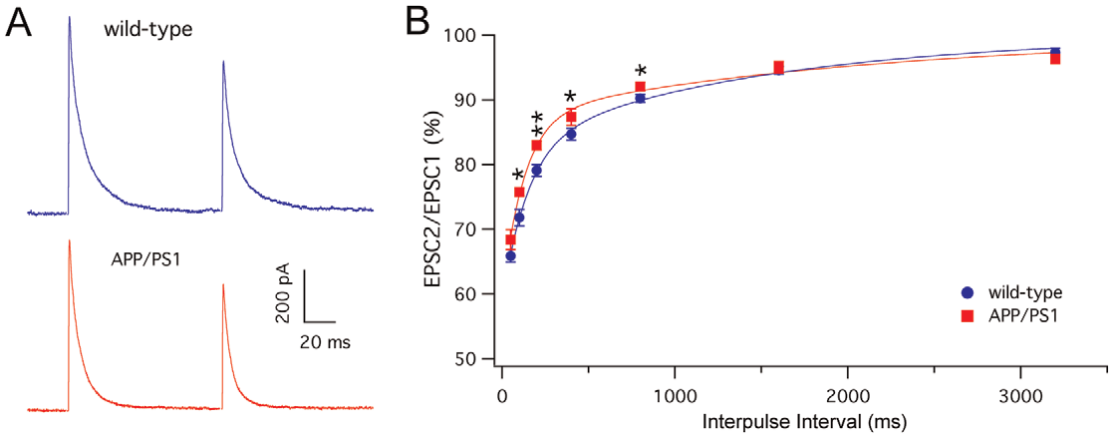
CF-EPSCs in wild-type and APP/PS1 PCs. (**A**) Representative traces of postsynaptic currents evoked by climbing fiber paired-pulse stimulation with an interpulse interval of 100 ms in wild-type (blue) and APP/PS1 (red) mice. (**B**) Time course of paired-pulse depression of CF-EPSC in wild-type (blue circles, n = 12) and APP/PSl mice (red squares, n = 10). The lines are double exponentials fittings of wild-type and APP/PS1 data points. The paired-pulse depression is expressed as the percentage of the amplitude of the second EPSC relative the first one (mean ± SEM) and is plotted as a function of interpulse intervals. (*p<0.05; **p<0.01, Student's t-test).

The amplitude of CF-EPSCs showed no significant difference between wild-type and APP/PS1 mice (Fig. 8A; wild-type: 0.94±0.11 nA, n = 12; APP/PS1: 1.49±0.50 nA, n = 10; Student's t-test, P>0.05). Short-term depression was analyzed by the paired-pulse protocol at interpulse intervals ranging from 50 to 3200 ms. At intermediate intervals, between 100 and 800 ms, APP/PS1 PCs displayed a reduced short-term depression relative to wild-type (Fig. 8B; Student's t-test, P<0.05). The time course of short-term depression was described by double exponential functions. APP/PS1 PCs, compared with wild-type, showed a higher proportion and a shortening of the fast time constant (wild-type: τ_f_ = 146.0 ms, 58.9%; APP/PS1: and τ_f_ = 125.8 ms, 69.3%), in line with the smaller depression at relatively brief intervals.

**Figure 9:**
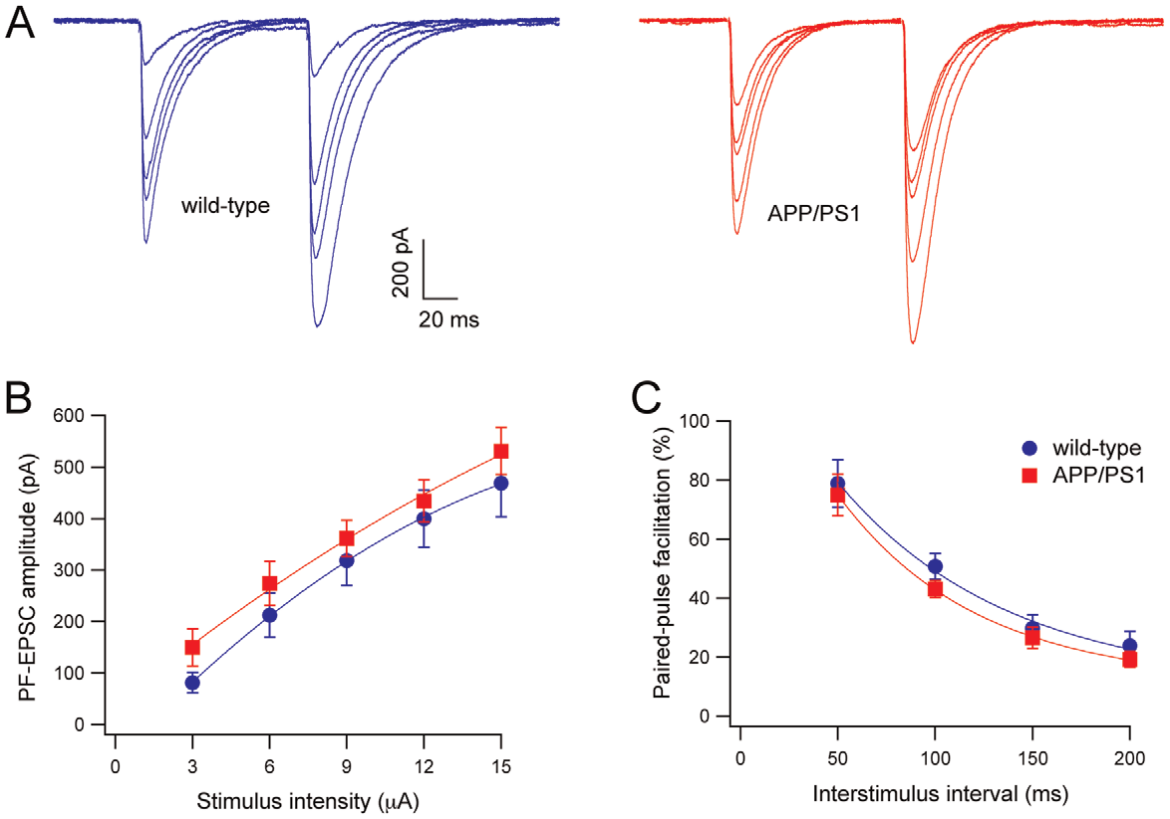
PF-EPSCs in wild-type and APP/PS1 PCs. (**A**) Representative traces of PF-EPSCs evoked by paired-pulse stimulation with an interpulse interval of 100 ms in wild-type (blue) and APP/PS1 (red) mice. Five traces obtained with stimulus strength from 3 to 15 µA are superimposed. (**B**) Amplitudes of PF-EPSCs are plotted as a function of stimulus intensity for wild-type (blue circles, n = 14) and APP/PS1 (red squares, n = 17) PCs. (**C**) Time course of paired-pulse facilitation of PF-EPSC in wild-type (blue circles, n = 17) and APP/PSl mice (red squares, n = 14). The facilitation is expressed as the percentage of the second EPSC relative to the first one (mean ± SEM) and is plotted as a function of interstimulus interval.

EPSCs evoked by parallel fiber stimulation (PF-EPSCs) were recorded at a V_H_ of −90 mV. The stimulating electrode was placed in a standard position in the middle of the molecular layer between the PC soma and the pial surface. Parallel fibers were stimulated with intensities ranging from 3 to 15 µA (Fig. 9A). The PF-EPSC amplitudes, at any intensity of stimulation, showed no significant difference between wild-type (n = 17 cells) and APP/PS1 (n = 14 cells; Student's t-test, P>0.05; Fig. 9B). Moreover, also the time course of paired-pulse facilitation was similar in wild-type and APP/PS1 PCs at all interpulse intervals (from 50 to 200 ms; Student's t-test, P>0.05; Fig. 9C). This result indicates that the PF-PC synapse is not altered in APP/PS1 mice.

Table 2 contains a list of the main synaptic parameters analyzed in wild-type and APP/PS1 PCs.

**Table 2:** Comparison of synaptic parameters of wild-type versus APP/PS1 mice of 8 months of age.

	evoked IPSC amplitude (pA)	evoked IPSC CV	evoked IPSC CV^−2^	miniature IPSC amplitude (pA)	miniature IPSC frequency (Hz)	Muscimol-evoked current (nA)	CF_EPSC amplitude (nA)	CF-EPSC PPD at 200 ms (%)	PF_EPSC amplitude at 9 µA (pA)
Wild-type 8 months	218.6±41.8 n = 10	0.48±0.06 n = 10	6.82±1.65 n = 10	73.8±7.0 n = 24	0.58±0.08 n = 24	1.52±0.14 n = 14	0.94±0.11 n = 12	79.1±0.9 n = 12	318.8±48.5 n = 17
APP/PS1 8 months	250.8±39.4 n = 11	0.42±0.03 n = 11	7.04±1.56 n = 11	68.9±4.9 n = 31	0.60±0.09 n = 31	1.48±0.18 n = 14	1.49±0.50 n = 10	83.0±0.7 n = 10	361.5±34.8 n = 14
Single data points *P* (Student's t test)	n.s.	n.s.	n.s.	n.s.	n.s.	n.s.	n.s.	<0.01	n.s.
Cumulative distribution *P* (Kolmogorov-Smirnov test)	-	-	-	<0.001	<0.001	-	-	-	-

## Discussion

In this study we show that, at the age of 7–8 months when numerous amyloid plaques are present in the forebrain, the cerebellum of APP/PS1 mice contains high levels of soluble Aβ_1–42_, which are associated with a reduction of membrane excitability of PCs and an altered GABAergic signaling. Furthermore, we show a reduction of paired-pulse depression at the CF-PC synapse. On the contrary, the function of the PF-PC synapse is spared.

It has been widely suggested that soluble, rather than fibrillar, Aβ is the most important pathogenic factor in AD [24]–[25]. Actually, the presence of low picomolar concentrations of soluble Aβ_1–42_ are necessary to enable synaptic plasticity in the hippocampus, but nanomolar doses impair hippocampal long-term potentiation [26]. Moreover, it has been shown that nanomolar doses of natural soluble oligomers of Aβ obtained from human cortex of AD patients are sufficient to induce neuronal alterations [27]. APP/PS1 mice are a murine model of AD, in which human mutated APP^Swe^ and PS1^L166P^ are produced under the control of a promoter mainly expressed in the forebrain [9]. However, soluble forms of Aβ can freely diffuse in the extracellular fluids and distribute in communicating compartments, including adjacent regions like forebrain and hindbrain [8]. Indeed we found that the levels of soluble Aβ_1–42_ in the cerebellum of APP/PS1 mice of 2 months of age are about half relative to the forebrain, but at 8 months the cerebellum contains about 40% more soluble Aβ_1–42_ than the forebrain. One possible explanation is that in the forebrain, where at 8 months of age the formation of amyloid plaques is massive, most of the Aβ peptide produced is being sequestered into plaques. In addition to the production of Aβ peptide, the high expression of the transgenes (the human *APP^Swe^* transgene has an expression of about three times that of endogenous mouse *APP* [9]) likely causes the generation of other APP fragments, including sAPPα, sAPPβ, AICD, CTFα, CTFβ. In this study the effects of these APP derivatives have not been tested. For this reason, we cannot exclude that some of the alterations described in this report are not due to Aβ but to other APP-derived fragments. However, since the transgenes are predominantly expressed in the forebrain, any effect on the cerebellar cortex should largely derive from diffusion between these two regions. Although diffusion of other extracellular fragments cannot be excluded, the most likely candidate for a diffusion sufficient to account for the effects observed on PC physiology is soluble Aβ. Another possible cause of electrophysiological alterations is the expression of PS1^L166P^, which has been shown to interfere with the release of Ca^2+^ from endoplasmic reticulum stores [28]. Alterations of ryanodine-dependent calcium release have been ruled out in our experiments on GABAergic IPSCs, but we cannot exclude different contributions to the control of membrane excitability.

Since PCs are the sole output of the cerebellar cortex, an alteration of their firing properties can be sufficient to disrupt the control on the target neurons in the deep cerebellar and vestibular nuclei. In fact, changes in the pattern or frequency of firing of PCs have important consequences also on the output from the deep cerebellar nuclei [29]. In our study, the deficit of excitability of PCs in the APP/PS1 mouse consists of a more pronounced frequency adaptation, with interspike intervals, which tend to increase in duration more than in control wild-type PCs. Such alteration might be due to the larger amplitude of the AHP following action potentials, because AHPs are known to be involved in delaying the subsequent firing of action potentials. This finding is in the same direction as the effect, in mouse dentate gyrus, of the application of synthetic Aβ_1–42_ oligomers, which in one study caused a reduction of neuronal excitability [30]. A second case, in which Aβ_1–42_ production was associated with a reduction of membrane excitability, was observed in 5XFAD mice, bearing 5 familial AD transgenes. In hippocampal CA1 pyramidal neurons of such 5XFAD mice, basal and learning-related excitability was reduced relative to control mice [31]. However, in contrast to these results, several studies found neuronal hyperexcitability in the hippocampal-entorhinal cortex network [19]–[21].

GABAergic evoked and miniature IPSCs in APP/PS1 mice have, on average, the same amplitude and frequency as in wild-type. However, the distributions of mIPSC amplitudes and of ISIs are significantly altered. In APP/PS1 mice, in the distribution histograms, the largest amplitudes and the longest intervals are underrepresented. An alteration of GABAergic signaling is in accordance with recent evidence that a GABAergic impairment may be important in the pathogenesis of network dysfunction in AD [32]. In fact, AD patients have decreased GABA or somatostatin levels in the brain and cerebrospinal fluid [32]–[37]. Furthermore, it has been recently reported that, in hAPP/PS1 mice, cerebral cortical neurons are hyperactive [22]. Such hyperactivity is associated with decreased GABAergic inhibition [22].

In cerebellar PCs, in addition to the classical role of voltage-gated Ca^2+^ channels in neurotransmitter release, it has been shown that the largest mIPSCs depend on a Ca^2+^-induced release of Ca^2+^ from intracellular stores [23]. Presenilin-1 mutations found in FAD patients have profound effects on cellular Ca^2+^ homeostasis [38]–[39]. The expression of FAD mutated presenilin, including the L166P mutation of our APP/PS1 mice, disrupts Ca^2+^ leak from intracellular Ca^2+^ stores [28], thereby causing an enhancement of Ca^2+^ release. In our recordings, PCs from APP/PS1 mice do not show an increase of large mIPSCs as expected by the effect of the mutated presenilin, but they present the opposite phenomenon, which is a reduction of large mIPCSs. However, in our experiments ryanodine application produced the same effects in APP/PS1 and wild-type PCs, indicating that the function of ryanodine receptors of the endoplasmic reticulum of cerebellar GABAergic interneurons was normal. The absence of the enhancement of Ca^2+^ release, which would be expected from the expression of PS1^L166P^, is in line with the fact that in our APP/PS1 mice the expression of the transgenes is low in the cerebellum. This finding strengthens the hypothesis that the functional alterations described in this study are due to diffusion of soluble factors like Aβ or other secreted products of APP^Swe^ from the forebrain rather than to the expression of the transgenes by cerebellar neurons.

Changes of the responsiveness of the postsynaptic membrane to GABA are also unlikely because the distribution of mIPSCs is not shifted and their mean amplitude is preserved in APP/PS1 mice. A lack of involvement of postsynaptic GABA_A_ receptors is confirmed by the fact that the application of the GABA_A_ agonist muscimol produces similar effects in wild-type and APP/PS1 mice. Taken together, our results on mIPSCs indicate that APP/PS1 mice have alterations of the axonal mechanisms regulating the release of GABA from cerebellar interneurons and that such defects are not due to a different contribution of Ca^2+^ induced Ca^2+^ release from ryanodine-sensitive intracellular stores.

In contrast to these alterations of the GABAergic synapses, the glutamatergic synapses formed by PF and CF are relatively intact. The transmission at the PF-PC synapse is completely normal, suggesting that, in this synapse, both presynaptic boutons and postsynaptic dendritic spines are functionally normal at qualitative and also quantitative levels. The amplitude of the CF-evoked EPSC was also normal, indicating that dendritic spines occupied by climbing fiber varicosities are likely to be functionally normal. The only functional alteration of glutamatergic synapses formed on PCs was a reduction of the paired-pulse depression of CF-EPSCs, which is considered as a presynaptic mechanism [40]. Therefore, the release of glutamate from the CF is altered, so that a second action potential in a time window between 100 and 800 ms is more efficient in APP/PS1 mice.

These alterations are likely to have complex consequences on the cerebellar cortical network. In APP/PS1 mice, PCs are less excitable, as they discharge fewer action potentials in response to depolarizing stimuli. In addition, they present minor alterations of the synapses formed by GABAergic interneurons and by CFs. The lack of large mIPSCs might correspond to a less efficient inhibitory action of stellate and basket cells on PCs. Indeed, a reduction of GABAergic efficiency would favour excitation, rendering the cells more easily driven towards threshold by excitatory inputs. In addition to this reduced GABAergic function, in APP/PS1 PCs, the excitatory CF-PC synapse is more powerful in a time window of 100–800 ms from a previous complex spike. These two effects could be additive, causing a tendency of APP/PS1 PCs to reach action potential threshold more frequently. Along this line, the decrease of intrinsic excitability could be envisioned as a compensatory mechanism, aimed at re-establishing the physiological rate of PC firing altered by the synaptic changes. However, at present it not possible to exclude the opposite alternative, that the synaptic alterations are compensatory for the impairment of intrinsic excitability. Future experiments are necessary to determine whether one of the two events is primary and the other is compensatory. The concept of a reciprocal compensation of membrane excitability and synaptic alterations is supported by the fact that an extensive series of behavioral tests showed a conserved motor performance and a lack of symptoms attributable to cerebellar dysfunction (Material S1).

## Materials and Methods

### Ethics statement

The animal experimental procedures were approved by the Bioethical Committee of the University of Turin (prot. 404 of June 17, 2005), have been communicated to the Ministry of Health (January 12, 2005 and October 21, 2008) and are in accordance with the European Union Directives 86/609/EEC and 6106/10/EU.

### Animals

Seven to eight months old APP/PS1 transgenic mice (n = 37) and their wild-type littermates (n = 35) of male gender were used for all the experimental paradigms. Evoked action potentials were studied also in 2 months old APP/PS1 mice (n = 3) and their wild-type littermates (n = 3). The APP/PS1 double transgenic mice (genetic background C57BL/6J) express mutated PS1 (PS1^L166P^) and APP (APP^Swe^, harboring the double KM670/671NL mutation) both under the control of a neuron-specific Thy1 promoter element [9]. Transgenic mice were obtained from Dr. Mathias Jucker, Hertie-Institute for Clinical Brain Research, University of Tübingen (Germany).

### Aβ ELISA

Total proteins of cerebella and forebrains from 2, and 8 month old mice (n = 3 for each age group) were extracted in Tris-buffered saline [150 mM NaCl, 50 mM Tris base, pH 8, 1% Triton X-100, protease inhibitor cocktail (Sigma Aldrich)] at 1 ml buffer/150 mg wet weight tissue. After centrifugation (25 min at 13,000 rpm at 4°C), the supernatant was used to measure soluble Aβ produced under the action of the human transgenes. The levels of soluble Aβ_1–42_ were quantified using the Innotest Aβ amyloid 1–42 high sensitivity test-ELISA kit (Innogenetics, Belgium). The kit uses an antibody that does not recognize mouse endogenous Aβ. Aβ_1–42_ levels were standardized to brain tissue weight and expressed as the ratio of the value in the cerebellum relative to the forebrain. The levels of soluble Aβ_1–42_, measured in serum from the same animals, were stable between the two ages analyzed and close to blank values.

### Slice preparation

Cerebellar slices were prepared as previously described [41]. The animals were anesthetized with isoflurane (Isoflurane-Vet, Merial, Italy) and decapitated. The cerebellar vermis was removed and transferred to an ice-cold artificial cerebrospinal fluid (ACSF) containing (in mM); 125 NaCl, 2.5 KCl, 2 CaCl_2_, 1 MgCl_2_, 1.25 NaH_2_PO_4_, 26 NaHCO_3_, 20 glucose, which was bubbled with 95% O_2_/5% CO_2_ (pH 7.4). Parasagittal cerebellar slices (200 µm thickness) were obtained using a vibratome (Leica Microsystems GmbH, Wetzlar, Germany) and kept for 1 h at 35°C and then at 25°C. Single slices were placed in the recording chamber, which was perfused at a rate of 2–3 ml/min with ACSF bubbled with the 95% O_2_/5% CO_2_. All recordings were performed at room temperature (22–25°C). Data of each experimental paradigm derive from 3 to 5 animals.

### Electrophysiology

Whole-cell patch-clamp recordings were made from PCs of adult animals using an EPC-9 patch-clamp amplifier (HEKA Elektronik, Lambrecht/Pfalz, Germany). Recordings were accepted only if the series resistance was less than 9.0 MΩ (range: 5.0–9.0 MΩ), and if it did not vary by >20% during the experiment. The soma of PCs was visually identified using a 40× water-immersion objective of an upright microscope (E600FN, Eclipse, Nikon, Japan), and its upper surface was cleaned by a glass pipette, pulled from sodalime glass to a tip diameter of 10–15 µm, containing the saline solution. Pipettes of borosilicate glass with resistances between 2.5 and 3.0 MΩ were used for patch-clamp recording. Patch pipettes were filled with an internal solution containing (in mM): 130 CsCl, 4 MgCl_2_, 10 HEPES, 4 Na_2_ATP, 0.4 Na_3_GTP, 10 EGTA, 5 N-(2,6-dimethylphenyl)acetamide-2-triethylammonium bromide (QX-314) and the pH was adjusted to 7.3 with CsOH and filtered at 0.2 µm. Cs^+^ blocks most outward currents through K^+^ channels while QX-314 blocks voltage-gated Na^+^ channels. Data were filtered at 3 kHz and sampled at 10 kHz. For all the experiments, digitized data were stored on a Macintosh computer (G3, Apple computer, Cupertino, CA, USA) using the Patch Master software (HEKA Elektronik, Lambrecht/Pfalz, Germany) and analyzed off-line.

#### Current clamp recordings

For current clamp recordings patch pipettes were filled with a K-gluconate-based internal solution containing (in mM); 140 K-gluconate, 10 HEPES, 0.5 EGTA, 4 MgCl_2_, 4 Na_2_ATP, 0.4 Na_3_GTP and the pH was adjusted to 7.3 with KOH and filtered at 0.2 µm. Gabazine (SR 95531, 20 µm) and kynurenic acid (1 mM) were added to the perfusate to inhibit the GABA_A_ and ionotropic glutamate receptors of PCs. Recordings were performed after manually adjusting the holding current at a value, which kept the membrane voltage close to −70 mV (±1 mV). Neurons in which the holding current was greater than 400 pA were discarded. Data were filtered at 8.6 kHz and sampled at 20 kHz. A series of current steps, each lasting 1000 ms, was delivered to the PC. Such current steps ranged from −400 to +1000 pA, in increments of 100 pA, with a step interval of 10 s. Data were analyzed using Axograph software (AxoGraph Scientific, Sydney, Australia) and afterwards the data were collected in a Microsoft Excel (Microsoft Corporation, Bellevue, WA, USA) spreadsheet.

#### Passive membrane properties characterization

In order to analyze the input resistance and the voltage bump due to the inward rectifier cationic current (I_H_), we measured the input resistance from the maximal negative deflections from the baseline, evoked by hyperpolarizing current steps ranging from −400 to −100 pA (five traces were measured for each point) while the amplitude of the voltage bump was measured as the difference between the peak negative deflection and the stable voltage level reached during the hyperpolarizing current step [42]. Such values were plotted as a function of the intensity of the respective hyperpolarizing currents, and the slope of the best fitting regression line was taken for each cell.

#### Active membrane properties characterization

Single action potential (AP) features were analyzed on traces evoked by the delivery to the PCs of a current step of +600 pA. All parameters of each cell were measured in five traces and averaged. The analyzed action potential properties were: threshold, AP amplitude, AP afterhyperpolarization (AHP), interspike interval (ISI). ISI was defined as the distance between the peaks of two consecutive APs. Threshold was measured in the first derivative of the AP (dV/dt) considering the point where the velocity was closest to 50 mV/ms. AP amplitude was measured as the voltage difference between the threshold and the absolute value reached at the peak. AHP amplitude was calculated as the voltage difference between the threshold and the negative AHP peak. Post–burst AHP (PB-AHP) was analyzed in responses to current steps of +1000 pA and it was calculated as the voltage difference between the baseline before the depolarizing step and the negative deflection after the action potential burst.

#### Responses of PCs to climbing fiber (CF) stimulation

To evoke excitatory postsynaptic currents (EPSCs) derived from CF (CF–EPSCs) inputs onto PCs, square pulses (100 µs) were applied through a stimulating electrode placed in the granular layer. CF-EPSCs were recorded at a holding potential of +40 mV to avoid the problems related to the large size of CF-evoked synaptic currents at negative potentials. CF-EPSCs were identified by their all-or-none fashion and the presence of paired pulse depression [43]–[44]. When the threshold was detected, the paired pulse depression was elicited by twin pulses at different time intervals (50, 100, 150, 200, 400, 800, 1600, 3200 ms). All CF recordings were performed in the presence of the GABA_A_ antagonist gabazine (SR 95531, 20 µM) in the saline solution.

#### Responses of PCs to parallel fiber (PF) stimulation (PF-EPSCs)

PF-EPSCs were evoked by stimulating the PFs in the molecular layer and recorded at a holding potential of −90 mV, to exploit the advantages of a larger distance from the reversal potential to obtain a better resolution of PF-evoked currents, and of keeping the voltage far from the threshold for most voltage-dependent conductances. Negative current pulses ranging from 3 to 15 µA with a duration of 100 µs were delivered at 20 s interval. Paired pulse facilitation was elicited by twin pulses of 9 µA of intensity at different time intervals (50, 100, 150, 200 ms), and the ratio of the amplitude of the second PF-EPSC over the first was calculated. All PF recordings were performed in the presence of gabazine (SR 95531, 20 µm) in the saline solution.

#### Inhibitory postsynaptic currents (IPSCs)

Miniature IPSCs (mIPSC) were recorded from PCs at a holding potential of −70 mV in the presence of the glutamate antagonists D- (-) –2-amino–phosphonopentanoic acid (D-AP5, 10 µm) and NBQX (20 µm), and of tetrodotoxin (TTX; 1 µm). The analysis of spontaneous and miniature events was performed with Mini Analysis software (Synaptosoft Inc., Decatur, GA, USA). mIPSCs with amplitudes <5 pA were discarded. Events with 10–90% rise times greater than 2 ms were also discarded. Mean mIPSC frequency and amplitude values were determined from at least three consecutive epochs of 50 s. Cumulative frequency plots were constructed by integrating the distribution histograms of mIPSC amplitudes and inter-event intervals.

### Drugs

Gabazine was purchased from Sigma Chemical (St. Louis, MO, USA). D-AP5, NBQX, kynurenic acid, muscimol and TTX were purchased from Tocris Cookson (Langford, UK). Ryanodine was purchased from Ascent Scientific Ltd (Bristol, UK). All drugs were applied via the chamber perfusion line.

### Statistics

Data are presented as mean value ± SEM. Unless otherwise indicated, n = number of cells. For data which passed the normality test, the statistical comparison was performed either by the paired or the unpaired two tailed Student's t-test. Data for which the normality test failed, were compared by the Mann-Whitney u-test. Cumulative frequency plots were analyzed by Kolmogorov-Smirnov (KS) test. All the graphs were designed using Igor Pro (WaveMetrics, Lake Oswego, Oregon, USA), and statistical tests were performed by means of SPSS software (SPSS Inc., Chicago, IL, USA). *P* values lesser than 0.05 was accepted as significant.

## Supporting Information

Material S1 **Wild-type and APP/PS1 mice were subjected to a set of motor tests.** In fixed bar test, footprinting test and beam test, there was no significant difference between the two groups. In the accelerated rotarod test there was no significant difference either for the initial performance or for the improvement over three consecutive days or for the retention test seven days later.
(DOC)
