# Supplementary material for: Excitability and Synaptic Alterations in the Cerebellum of APP/PS1 Mice
Source: PLoS One. 2012 Apr 12;7(4):e34726. doi: 10.1371/journal.pone.0034726 (PMC3325253; doi:10.1371/journal.pone.0034726)
Supplement: Material S1 — Wild-type and APP/PS1 mice were subjected to a set of motor tests. In fixed bar test, footprinting test and beam test, there was no significant difference between the two groups. In the accelerated rotarod test there was no significant difference either for the initial performance or for the improvement over three consecutive days or for the retention test seven days later. (DOC) [file pone.0034726.s001.doc]

**Supporting Material S1**

**Motor tests**

To assess whether the alterations of synaptic transmission are accompanied by impairment of cerebellar dependent motor tasks, wild-type and APP/PS1 mice were subjected to a set of behavioral tests. The fixed bar test is aimed at probing strength and motor coordination. In this test, the latency of falls was not significantly different in APP/PS1 mice relative to wild-type (Suppl. Table). In a variant of the footprinting test, subtle parameters related to even mild ataxic gait, like stride variation and forepaw/hindpaw placement distance, have been analyzed. Even these parameters were not different in the two groups of animals (Suppl. Table), indicating intact gait mechanisms and absence of cerebellar gait ataxia. The beam test evaluates the motor coordination and balance, which are also dependent upon cerebellar function. Also in the beam test no difference was detected between APP/PS1 and wild-type mice. Repetition of the beam test in the following two days normally reveals a significant improvement, which can be considered as sensory-motor learning. Such improvement was not significantly different in the two groups of animals, indicating that also learning capabilities are preserved in APP/PS1 mice.

The accelerated rotarod test was used as an additional evaluation of motor coordination and learning. In the initial performance there was no difference between wild-type and APP/PS1 mice (t-test, P > 0.05; Suppl. Fig). With 3 training sessions per day for three consecutive days, both groups of animals showed a significant daily improvement (one way ANOVA: P < 0.001; Suppl. Fig). The statistical analysis did not reveal any significant difference between genotypes in any of these paradigms (two way ANOVA P > 0.05). After 7 days of rest, the mice were tested for retention. There was no significant loss of performance in either group (paired t-test, P > 0.05) and also in this case these was no difference between wild-type and APP/PS1 mice (two way ANOVA, P > 0.05).

**Supplementary Methods**

**Motor tests**

The fixed-bar test [S1] was performed to evaluate the balance and general motor function of the mice (n = 13 wild-type; n = 14 APP/PS1). The mice were placed in the middle of a horizontally held wood bar (round section 1 cm of diameter and 80 cm long) suspended 50 cm above an open cage. The time required for the animal to fall from the bar was recorded for a maximum of 120 s. Each animal was administered three trials.

A modified footprinting test was performed to evaluate the motor coordination and gaiting of the mice (n = 13 wild-type; n = 14 APP/PS1), as in Kalume et al. [S2]. Briefly, a cardboard tunnel was constructed on a transparent plexiglass platform (4 cm wide, 40 cm long). The tunnel, which was elevated above the bench, ended with a dark box. After allowing the mice to traverse the tunnel once (habit), video recordings of the mouse walking were collected by means of a digital camera placed underneath. The stack of frames produced by the video-camera was analyzed, frame by frame, by means of the ImageJ software. Data concerning stride length and variation (coefficient of variation), stride width and distance between ipsilateral forepaw and hindpaw placements were obtained.

The beam test was used to evaluate motor coordination, balance and short-term motor memory in transgenic (n = 14) and control (n = 13) mice. It was performed as described [S3]. Mice were placed at the end of an 80 cm-long wood beam (2 cm wide), suspended above an open cage. After allowing the mice to traverse the beam twice (habit), all lateral slips were counted in five complete and consecutive crossings per day on three consecutive days. The mean number of slips per 80 cm traveled was calculated.

Accelerating rotarod test [S4] was used in order to study motor coordination and motor learning. Mice (n = 23 wild-type; n = 23 APP/PS1), were tested for three consecutive days and then after 7 days (10^th^ day). In each day, after a 2 minute training session at a constant speed (4 rpm), mice received three test sessions in which the rod (Mouse Rota-Rod, Ugo Basile Biological Research Apparatus, Comerio, Italy) accelerated continuously from 4 to 65 rpm over 350 s. The latency to fall off the rod was recorded.

**Supplementary References**

S1. Wirths O, Breyhan H, Schäfer S, Roth C, Bayer TA. Deficits in working memory and motor performance in the APP/PS1ki mouse model for Alzheimer's disease. Neurobiol Aging 2008;29:891-901.

S2. Kalume F, Yu FH, Westenbroek RE, Scheuer T, Caterall WA. Reduced sodium current in Purkinje neurons from Nav1.1 mutant mice: Implications for ataxia in severe myoclonic epilepsy in infancy. J Neurosci 2007;27:11065-74.

S3. Chen X, Kovalchuk Y, Adelsberger H, Henning HA, Sausbier M, Wietzorrek G, Ruth P, Yarom Y, Konnerth A. Disruption of the olivo-cerebellar circuit by Purkinje neuron-specific ablation of BK channels. Proc Natl Acad Sci U S A 2010;107:12323-8.

S4. Bearzatto B, Servais L, Cheron G, Schiffmann SN. Age dependence of strain determinant on mice motor coordination. Brain Res 2005;1039:37-42.

**Supplementary Figure**


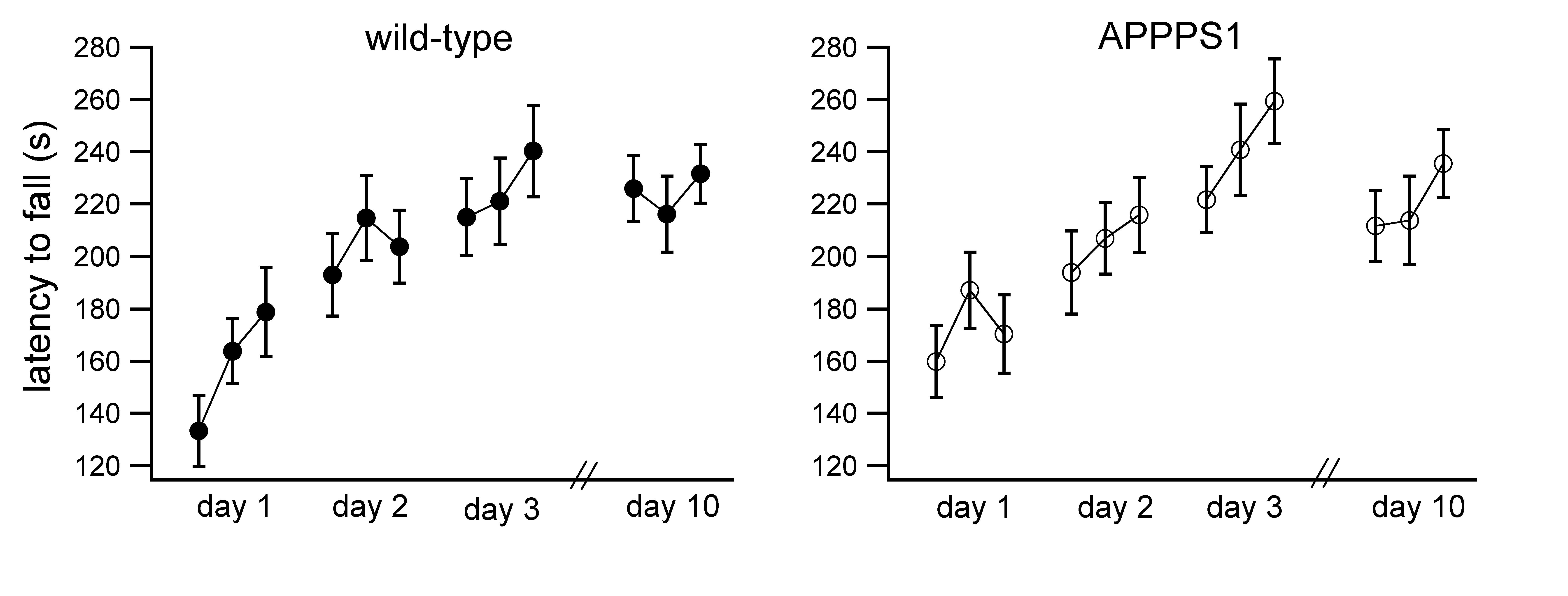


**Motor performance and learning in the rotarod test.** The latency to fall is plotted for each group of mice, for 3 daily trials repeated for three consecutive days and again on the 10^th^ day. Left panel: wild-type mice; right panel: APPPS1 mice. Bars are S.E.M. (n = 23 for both groups).

**Supplementary Table. Performance of wild-type and APPPS1 mice in motor tests.**

| ***Behavioral test*** | ***Motor performance parameters*** | ***Values (±SE)*** | ***Statistical difference*** |
| --- | --- | --- | --- |
| Fixed bar test | Strength and motor coordination  (latency) | wt: 59.7±12.4s;  APPPS1: 65.3± 12.1s | n.s. |
| Footprinting test | Stride length | wt: 5.7±0.3cm;  APPPS1: 6.3±0.2 cm | n.s. |
|  | Stride variation (CV) | wt: 0.3±0.02;  APPPS1: 0.2±0.02 | n.s. |
|  | Motor coordination  (forepaw-hindpaw distance) | wt:1.8±0.2cm;  APPPS1: 1.9±0.1cm | n.s. |
|  | Stride width | wt: 3.5±0.1cm;  APPPS1: 3.7±0.1cm | n.s. |
| Beam test | Motor coordination and balance  (mean number of slips/traveled distance in the 1^st^ day) | wt: 0.6±0.2;  APPPS1: 0.8±0.2 | n.s. |
|  | Short-term motor memory  (mean number of slips/traveled distance in the 2^nd^ and 3^rd^ days) | 2^nd^ day:  wt: 0.2±0.1;  APPPS1: 0.1±0.04  3^rd^ day:  wt: 0; APPPS1: 0 | n.s. |
